# Optimization of Diffusion-Ordered NMR Spectroscopy Experiments for High-Throughput Automation in Human Metabolic Phenotyping

**DOI:** 10.1021/acs.analchem.2c04066

**Published:** 2023-01-31

**Authors:** Nikita Harvey, Panteleimon G Takis, John C Lindon, Jia V Li, Beatriz Jiménez

**Affiliations:** † Section of Bioanalytical Chemistry, Division of Systems Medicine, Department of Metabolism, Digestion and Reproduction, Imperial College London, Burlington Danes Building, Hammersmith Hospital Campus, London W12 0NN, U.K.; ‡ National Phenome Centre, Department of Metabolism, Digestion and Reproduction, Imperial College London, phenomecentre@imperial.ac.uk, IRDB Building, Hammersmith Campus, London W12 0NN, U.K.; § Section of Biomolecular Medicine, Division of Systems Medicine, Department of Metabolism, Digestion and Reproduction, Imperial College London, Burlington Danes Building, Hammersmith Hospital Campus, London W12 0NN, U.K.; ∥ Section of Nutrition, Division of Digestive Diseases, Department of Metabolism, Digestion and Reproduction, Imperial College London, Commonwealth Building, Hammersmith Hospital Campus, London W12 0NN, U.K.

## Abstract

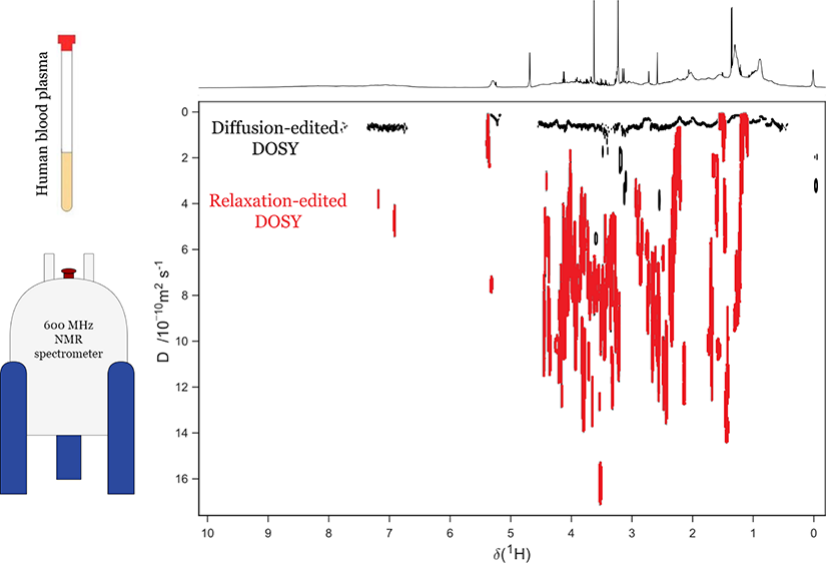

The diffusion-ordered nuclear magnetic resonance spectroscopy (DOSY) experiment allows the calculation of diffusion coefficient values of metabolites in complex mixtures. However, this experiment has not yet been broadly used for metabolic profiling due to lack of a standardized protocol. Here we propose a pipeline for the DOSY experimental setup and data processing in metabolic phenotyping studies. Due to the complexity of biological samples, three experiments (a standard DOSY, a relaxation-edited DOSY, and a diffusion-edited DOSY) have been optimized to provide DOSY metabolic profiles with peak-picked diffusion coefficients for over 90% of signals visible in the one-dimensional ^1^H general biofluid profile in as little as 3 min 36 s. The developed parameter sets and tools are straightforward to implement and can facilitate the use of DOSY for metabolic profiling of human blood plasma and urine samples.

Metabolic phenotyping provides a comprehensive snapshot of the metabolic content of biological samples.^1^ Typically, an untargeted metabolic profiling approach is used in which a general profile reflecting the chemical composition of the sample is obtained, allowing differences between groups of samples to be discovered and providing insight into disease-associated metabolic pathways. Therefore, metabolic profiling has a wide range of applications to biomedical research and epidemiological and clinical studies,^2^ with implications for predictive medicine.^3^

NMR spectroscopy is one of the primary methodologies used in metabolic profiling, being easily automated, robust, and highly reproducible.^4^ It is also versatile, allowing for multiple different assays to be performed on a unique sample by the same instrument. Three assays, namely, a one-dimensional (1D) ^1^H general profile, a 1D CPMG (Carr-Purcell-Meiboom-Gill), and a two-dimensional (2D) *J*-resolved experiment, are commonly run on large sample sets.^5,6^ These can each be acquired within 4 min, allowing for a high throughput of samples (60–80 samples typically analyzed within 24 h) thereby reducing sample degradation and making the assays cost-effective. While the majority of NMR-based metabolic profiling studies focus on measuring relative concentrations of metabolites, NMR spectroscopy can be used to assess other molecular properties in mixtures such as relaxation times or diffusion coefficients. The diffusion coefficient is a physical constant, representing the random passive Brownian motion of a substance, which depends on its size and interaction with its environment. The use of this constant in metabolic phenotyping could provide valuable information on the sample matrix properties. To this aim the 2D ^1^H diffusion-ordered NMR spectroscopy (DOSY) experiment has been optimized for metabolic phenotyping of biofluids.^7^

Thus far, the 2D DOSY experiment has not been attempted in a high-throughput analysis of large sample sets or been widely used to analyze human biofluid samples due to the lack of a standardized implementable pipeline. Biofluids are mixtures of variable complexity that require tailored experiments in order to increase the information that one can obtain from them. The aim of this work was to demonstrate a set of optimized DOSY experiments suitable for high-resolution and high-throughput analyses of biological samples and develop a workflow for the extraction of diffusion coefficient constants for the main components of these biofluids. Different experiments have been optimized to suit different biological samples and to exploit the DOSY properties such that both small and macromolecules can be analyzed.

## Experimental Section

Development and optimization of DOSY NMR methods for biofluids was done using pooled human biofluid samples, which were prepared according to published protocols.^6^

Experiments were carried out on spectrometers equipped with a Bruker AVANCE III 600 console with a 14.1 T magnet for ^1^H 600 MHz, a SampleJet sample handling robot, and a 5 mm inverse broadband (BBI) configuration probe with a *z*-axis magnetic field gradient capability. Data was acquired using TopSpin version 3.6 (Bruker) in automation. Each spectrometer was calibrated to ensure accurate measurements and reproducibility across different spectrometers. Data was acquired at either 300 or 310 K (±0.05 K) to keep consistency with the temperature used for high-throughput profiling of urine and blood plasma samples, respectively.^6^ Bruker pulse sequences (*noesygppr1d* and *ledbpgppr2s*) and automation methods/software were used in data acquisition. Standard metabolic profiling experiments were used (Supporting Information (page S2), and the setup was arranged as previously described,^5,6^ including automated acquisition/processing. For all samples, the *noesygppr1d* pulse sequence was used to produce a general 1D ^1^H NMR profile. Pulse lengths and center frequency were optimized per sample using the *noesygppr1d* experiment and transferred to DOSY experiments.

**Figure 1:**
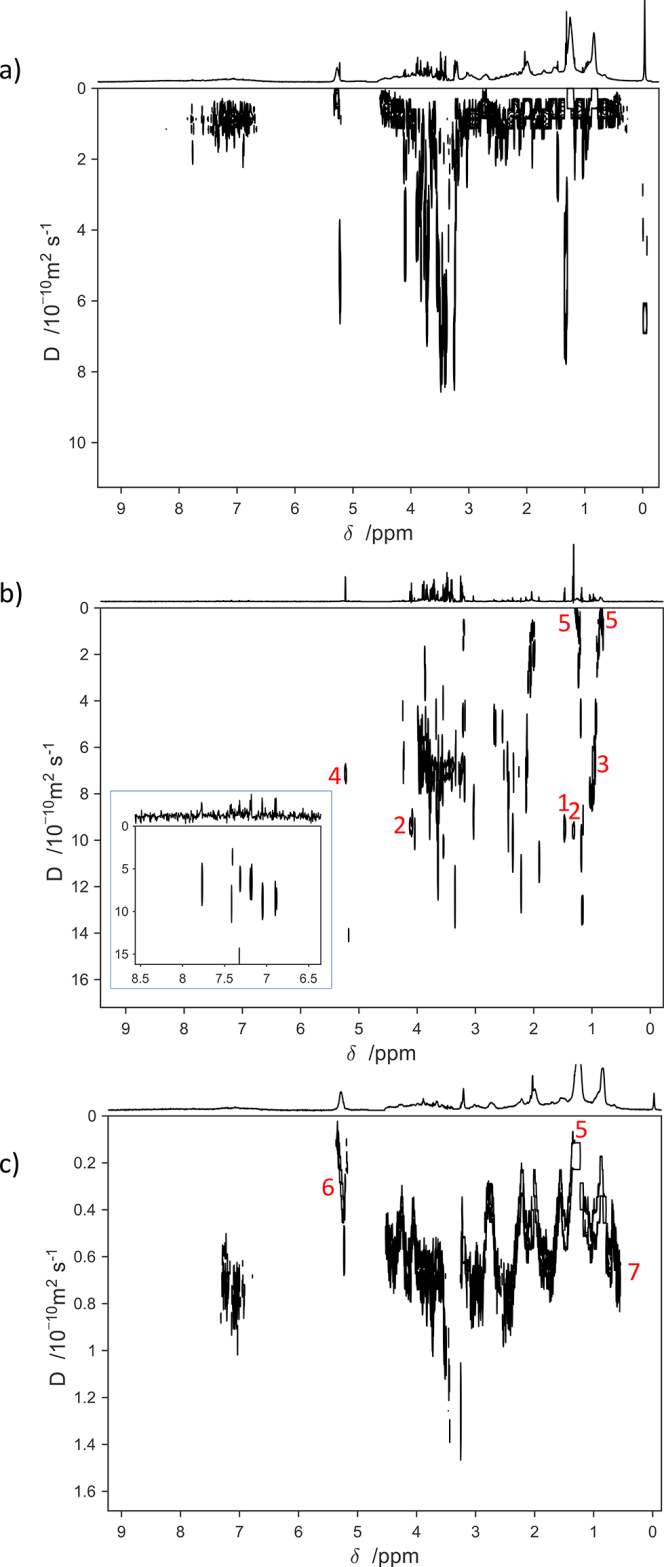
DOSY spectra of a pooled human blood plasma sample run using (a) the traditional DOSY experiment, (b) the relaxation-edited DOSY experiment, with an inset of the aromatic region at a different contour level, and (c) the diffusion-edited DOSY experiment. Several metabolites with high peak intensities are labeled in red in the relaxation-edited DOSY: (1) alanine, (2) lactate, (3) valine, (4) glucose anomeric peak, (5) lipoproteins. Further plasma components are labeled in the diffusion-edited DOSY: (5) lipoproteins, (6) lipids, (7) proteins. Above each 2D spectrum the internal 1D projection is shown. Diffusion coefficients of labeled metabolites are listed in Supporting Information, page S13.

The standard Bruker pulse sequence *ledbpgppr2s* was optimized for biofluids that do not contain macromolecules (Figure 1a).^8^ This has the form RD-90°-τ-180°-τ-90°-Δ-90°-τ-180°-τ-90°-D_e_-90°-ACQ, where 90° represents a 90° radiofrequency (RF) pulse, 180° represents a 180° RF pulse, RD is the relaxation delay of 2 s, τ is a short delay typically of about 3 μs, Δ represents the diffusion delay, D_e_ is the second eddy current delay of 5 ms, and ACQ is the data acquisition period. The spoil gradient pulse length was set to 600 μs. Gradients had a Gaussian shape, including two spoil gradients used for removing any transverse magnetization, which were −17.13% and −13.17% of the main gradient pulse. The gradient ramp used to calculate diffusion coefficient values was set with limits of 5% and 95% to reduce interference from the water signal.

For the small molecule DOSY analysis of blood plasma samples (Figure 1b), the PROJECTED (Periodic Refocusing of J Evolution by Coherence Transfer Extended to DOSY)^9^ sequence was modified to include a presaturation pulse for water suppression. This pulse sequence has the form of RD-90°-τ-180°–90°-τ-180°-τ-[τ-180°-τ-90°-τ-180°-τ]_*n*_-τ-180°-τ-90°-τ-180°-τ-ACQ with abbreviations as defined above, where *n* was set to 14 to provide relaxation editing. Six sets of gradient pulse pairs (gradient pulse length optimized to 800 μs) were used with each separated by the unit diffusion time Δ. The gradient ramp used to calculate diffusion coefficient values was set with limits of 5% and 95% to reduce interference from the water signal. See Supporting Information (page S4) for a modified pulse sequence for Bruker TopSpin 3.

The diffusion-edited DOSY experiment used in Figure 1c also used the *ledbpgppr2s* pulse sequence as described above. Full sets of optimized parameters for all three experiments are given in the Supporting Information (page S7).

Modified versions of the Bruker acquisition AU program *au_dosy* and an *ad-hoc* processing file were created to run and process the experiments in automation. These are provided in the Supporting Information (page S12).

DOSY spectra were generated using the General NMR Analysis Toolbox (GNAT).^10^ The 1D increments were processed with 64 k points, apodized with a Gaussian function (gw = 1), and phased; the water peak was pruned, and a noise threshold was set. Plasma spectra were calibrated by setting the chemical shift of the anomeric proton resonance of glucose to 5.23 ppm. GNAT was used to produce the fitted DOSY spectrum along with a peak-picked list of diffusion coefficients.

**Figure 2:**
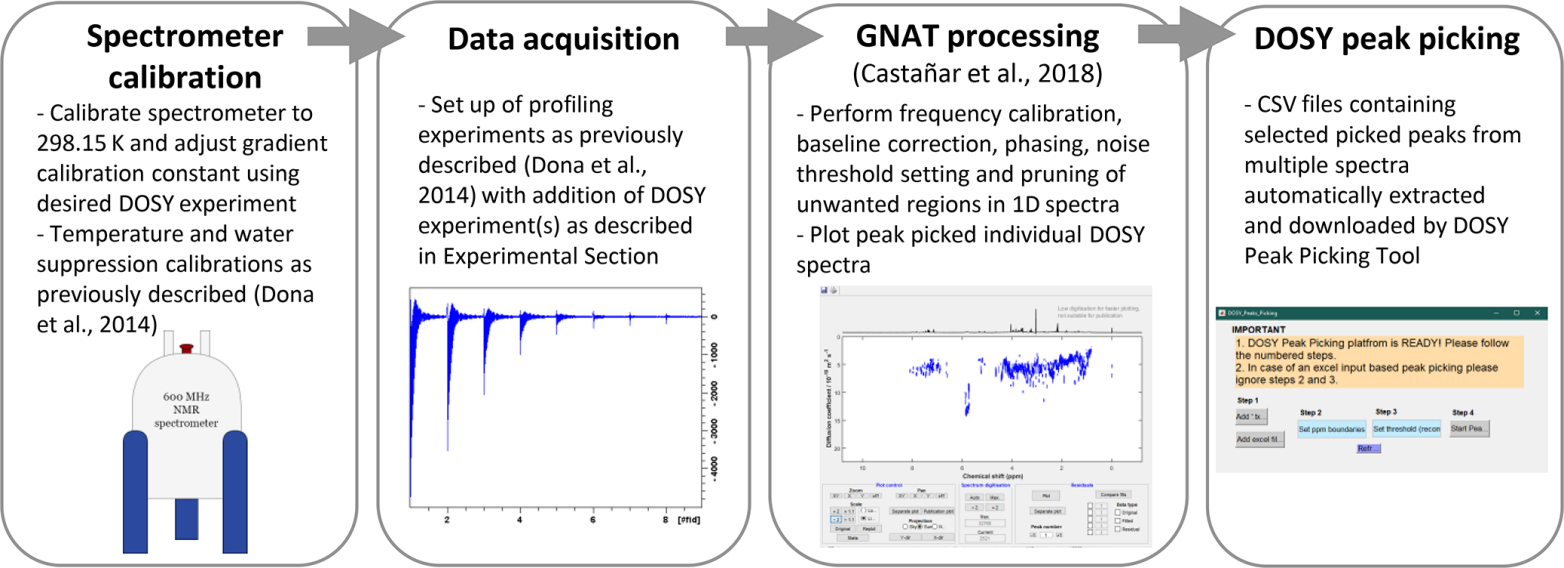
Suggested pipeline for running and processing DOSY spectra for metabolic profiling. Gradient calibration must be performed at 298.15 K using a “doped water” sample (1% H_2_O in D_2_O + 1 mg/mL GdCl_3_) to give a H_2_O diffusion coefficient of 1.91 × 10^–10^ m^2^/s using the desired DOSY pulse sequence. A presaturation power of 0 W should be used to avoid suppression of the water signal. The spectrometer must be calibrated as previously described^6^ prior to data acquisition. Spectra may be individually processed using GNAT^10^ to produce a list of picked DOSY peaks. Diffusion coefficients may then be extracted from the lists for multiple spectra for a specific chemical shift range using the DOSY peak picking tool.

An in-house graphical user interface software called DOSY Peak Picking was developed in the MATLAB 2019b programming environment. This software semiautomatically picked peaks in multiple spectra from a collection of lists based on chemical shift and fitting error provided by GNAT for each spectrum. This program performed automatic extraction of DOSY peak positions (i.e., peak picking function) for multiple spectra and significantly accelerated the spectral analysis (see Figure 2). CSV files containing selected picked peaks filtered by chemical shift and noise were automatically extracted and downloaded to allow one to compare diffusion coefficient values across multiple samples for specific chemical shift ranges. The software is freely available in the GitHub repository (https://github.com/pantakis/DOSY-NMR_Peaks_Picking).

## Results and Discussion

A standard DOSY pulse sequence including bipolar gradient pulse pairs, a longitudinal eddy current delay, and a stimulated echo sequence incorporating a diffusion delay^8^ was used for experimental optimization on human urine samples. To obtain diffusion coefficient resolution, the signals of interest should decay over the course of the experiment from 95% intensity in the first increment to ∼5% intensity in the final increment. The decay curve for each signal is fitted to the Stejskal-Tanner equation to obtain the diffusion coefficient. This signal decay is dependent on both the diffusion delay and the gradient pulse length. The diffusion delay (allowing molecules to diffuse in solution before further pulses are applied) was set to 50 ms, i.e., the shortest recommended^11^ delay length for the pulse sequence. It was found that a reduction in the diffusion delay from 75 to 50 ms increased the resolution in the diffusion dimension 4-fold allowing more accurate calculation of diffusion coefficients. This was due to the higher diffusion delay producing an overly steep signal decay curve. The gradient pulse length was adjusted to 1.5 ms to provide an optimal^11^ signal decay curve, with the initial increment giving a signal-to-noise ratio (SNR) of 681 on the deuterated TSP (3-(trimethylsilyl)-2,2,3,3-tetradeuteropropionic acid or TMSP-d4) signal and the final increment giving an SNR of 20. This provides a signal decay of 97.2%, showing that the experiment captures the fullest extent of the signal decay possible while allowing every increment to contribute to the fit of the decay curve for each signal.

Reduction of the number of scans and increments from 64 and 256, respectively, down to 8 and 8 gave a 228-fold decrease in experimental time, eliminated artifacts caused by signal decay fitting errors, and retained sufficient sensitivity to obtain peak-picked signals for 90% of the signals peak picked in the standard 1D ^1^H urine spectrum. This short experiment (3 min 36 s) allowed the spectroscopic separation of nonproteinaceous aqueous solutions of small molecules (Supporting Information, page S13), making it suitable for high-throughput human biofluid profiling where prevention of sample degradation and reduction of experimental time are key. A 32-scan experiment (∼13 min) resolves all signals visible in the standard 1D ^1^H spectrum and is therefore suitable if further sensitivity is needed.

While convection is often considered an issue in the acquisition of DOSY experiments, it was found that Rayleigh-Bénard convection does not occur to a significant degree in room-temperature probes and thus does not materially affect the diffusion coefficient measurements in these DOSY experiments (see Supporting Information, page S16).

This method was applied to a simple mixture of metabolites to observe their separation in the spectrum by diffusion coefficient (see page S13). The same experiment was then applied to a more complex biofluid mixture in the form of a human urine sample with individual metabolite signals identifiable. Diffusion coefficients were extracted for the labeled metabolites (see Supporting Information, page S13).

In blood plasma and other proteinic biofluids, the macromolecular proton envelope produces broad signals, which obscure the small metabolite peaks in the DOSY spectra. Figure 1a shows a DOSY spectrum of a pooled human blood plasma sample, run using the standard DOSY experiment. As the diffusion coefficient is calculated using peak intensity decay, the calculated diffusion coefficient of overlapping peaks is an average of those of the molecules contributing to the signal. This means that the broad peaks from the macromolecules obscure the signal decay of the small metabolite peaks making it difficult to obtain accurate metabolite diffusion coefficient values. Similarly, the macromolecule signals in the DOSY spectrum are disrupted by the overlapping small metabolite signals. Hence, two complementary experiments have been developed to analyze the small molecules (Figure 1b) and macromolecules (Figure 1c) separately from each other.

The most common method of removing macromolecular signals from biofluid NMR spectra is relaxation editing, most notably using the 1D CPMG spin–echo experiment, which is frequently used in the metabolic profiling analysis of blood plasma and serum samples.^6^ In the spectrum shown in Figure 1b, the PROJECTED^9^ pulse sequence was used to filter out the signals from macromolecules in the same human blood plasma sample using relaxation editing. This pulse sequence was selected due to the implementation of the double spin–echo sequence providing a spectrum free of *J*-modulation and chemical exchange effects. However, due to the limitations of the DOSY experiment, any overlapping signals within these separate DOSY spectra will still be subject to averaging of diffusion coefficients. These limitations are not unique to DOSY and are generally solved by acquiring additional experiments.

Optimization was performed on the PROJECTED experiment to adapt it for analysis of human blood plasma samples. A presaturation pulse was introduced into the pulse sequence to suppress the large water signal. The diffusion delay was optimized to compromise between editing out the macromolecule signals and minimizing noise (see Supporting Information, page S7). The SNRs of the signals observed in the first increment of the experiment (using 2% gradient strength) were used to quantify this optimization. The SNR of the alanine doublet at 1.4 ppm (chosen as a representative small molecule) was 1.54 times higher with a unit diffusion time (Δ) of 30 ms than with Δ = 40 ms, thereby giving a reduction in noise. The SNR of the macromolecule signal was 4.54 times smaller with Δ = 30 ms than with Δ = 20 ms, therefore providing more efficient macromolecule editing. The gradient pulse length was set to 0.8 ms to give the optimal^11^ signal decay curve—as an example, the glucose anomeric signal at 5.23 ppm decayed by 91.5% over the course of the experiment. Other parameters were set according to previously established metabolic profiling conventions. As with the traditional DOSY experiment, the numbers of scans and points were both set to 8 (4 min 5 s per sample). It was found that the 8-scan experiment retained 75% of the signals in the standard 1D CPMG experiment, while the 32-scan experiment retained 99% of the signals. The narrow peaks from small molecule signals are clearly identifiable in Figure 1b and correspond well to the 1D ^1^H (1D nuclear Overhauser effect spectroscopy (NOESY)) profile (see Supporting Information, page S2). While Aguilar et al. suggested a modification to the pulse sequence for convection compensation,^9^ this was found to make no difference to the diffusion coefficient values obtained in our study (Supporting Information, page S16), and a gradient calibration was found to be a more effective way to compensate for convection. Further details on experimental optimization methods are given in Supporting Information, page S7.

Individual metabolites are identifiable in the human blood plasma PROJECTED spectrum in Figure 1b. Labeled as examples are the alanine doublet at 1.47 ppm (1), the lactate doublet at 1.3 ppm and quartet at 4.14 ppm (2), valine doublets at 0.98 and 1.03 ppm (3), the glucose anomeric proton doublet at 5.23 ppm (4), and lipoprotein signals at 0.84 and 1.25 ppm (5). Average diffusion coefficient values for these components are listed in Supporting Information, page S13, and are clearly different than those for the same metabolites in the other solutions (urine and the simple metabolite mixture). As expected, the glucose signals across the PROJECTED spectrum show a consistent diffusion coefficient ((7.20 ± 0.2) × 10^–10^ m^2^/s). However, in the traditional DOSY spectrum (Figure 1a), these signals are spread across a much wider range ((6.0 ± 1.2) × 10^–10^ m^2^/s) in the diffusion plane due to the overlap with the protein proton envelope. This demonstrates the importance of relaxation editing for obtaining accurate small molecule diffusion coefficients.

The complementary method used to observe diffusion coefficients of macromolecules in blood plasma samples was an adaption of the traditional DOSY experiment used for nonproteinic mixtures to incorporate diffusion editing. A much longer diffusion delay of 350 ms was implemented into the DOSY experiment, such that not only the small molecule signals decayed over the course of the experiment but also the macromolecule signals. This gave a protein signal decay of 84.2% with the final increment at 95% gradient compared to the first increment at 2%. As the small molecule signal decay occurred over the first 25% of increments, the gradient ramp used to calculate diffusion coefficient values was set with limits of 25% and 95%. This meant that the first increment of the diffusion-edited experiment had minimal small molecule signal (5.5% of the full signal) and retained the majority of macromolecule signal (82.2% of the full protein signal), while the final increment retained its almost complete signal decay. Experiments were run in 14 min 31 s per sample.

Different classes of molecules can be distinguished in Figure 1c. Protein signals including regions around 2.5, 2.9, and 3.7–4.1 ppm and the aromatic region (marked 7) are visible around (0.7–0.85) × 10^–10^ m^2^/s. The broad signals from lipids at 5.15–5.4 ppm are spread across a diffusion coefficient range of (0.01–0.45) × 10^–10^ m^2^/s. It is notable that the lipoprotein signals (marked 5) are visible in all three spectra in Figure 1 and that their signal decay is incomplete in the diffusion-edited spectrum, as their diffusion coefficients are less than 0.1 × 10^–10^ m^2^/s (see Supporting Information, page S13). In order to obtain a full decay of the lipoprotein signal a diffusion delay of over 700 ms is required, which would require doubling the number of increments and still does not resolve lipoprotein subclasses. This suggests that further optimization is required for the detailed analysis of lipoproteins.

For the application of the optimized DOSY experiments to the standard metabolic profiling workflow, the following procedure is proposed as laid out in Figure 2. Prior to any study setup, the gradient calibration of a spectrometer must be optimized for each DOSY pulse sequence to be used. The temperature and performance of the spectrometer should then be calibrated and samples run using standard metabolic profiling protocols as previously described^6^ with the addition of the DOSY experiment setup as described in the Experimental Section. Each DOSY spectrum may then be read into GNAT and processed as described to obtain a full list of picked peaks with diffusion coefficients in text format.

GNAT output files contain a large number of calculated diffusion coefficients along with the fitting errors per picked peak. Many of these peaks correspond either to artifacts or low-intensity signals that provide diffusion coefficients with high uncertainty. Thus, we constructed a freely available software in the MATLAB 2019b programming environment, incorporated into a graphical user interface (GUI), called DOSY Peak Picking. It allows the user to select a threshold of fitting errors and a range of picked peaks in multiple spectra from the GNAT outputs, to automatically extract only the highly reliable calculated diffusion coefficients of the picked peaks. In addition, it allows the user to focus on a specific or multiple spectral regions. The output of DOSY Peak Picking consists of a CSV file containing selected picked peaks filtered by chemical shift and diffusion coefficients fitting error for all spectra. More details are included in the user guidelines at the GitHub repository. Consequently, our software significantly accelerates the DOSY spectra analysis, allowing the smoother incorporation of DOSY into a more automated and user-friendly “-omics”-like pipeline (Figure 2). The DOSY Peak Picking tool may therefore be used to extract diffusion coefficient data from multiple spectra for specific metabolites, allowing one to have a targeted metabolomics analysis based on diffusion coefficient.

## Conclusions

We propose a pipeline for the incorporation of the DOSY experiments into routine metabolic profiling of human biofluids. With the correct optimization and the development of tools, the DOSY experiments have clear potential to be used in metabolic profiling. An optimized DOSY experiment that provides 90% of signals in 3 min 36 s has been demonstrated for urine. A complementary pair of DOSY experiments based on relaxation and diffusion editing have been shown to work well for plasma/serum samples and together provide data for over 75% of the signals in less than 20 min. A peak picking tool has been developed to automate the processing of multiple spectra at the same time. These experiments require a short acquisition time, making them suitable for the analysis of cohorts of clinical samples. The sizes of these cohorts could be limited by the necessity of processing each DOSY spectrum individually, and high-quality automated tools should be developed to facilitate the processing of large batches of DOSY spectra.

## Supporting Information Available

1: Pulse Sequences used for the 1D Analysis of Blood Samples; 2: Modified PROJECTED pulse sequence file for Bruker TopSpin; 3: Modified PROJECTED pulse sequence file for Bruker TopSpin; 4: Automated acquisition and processing files for Bruker TopSpin; 5: Measured Metabolite Diffusion Coefficient Values; 6: Convection in the DOSY experiments (PDF)
