# Supplementary material for: Optimization of Diffusion-Ordered NMR Spectroscopy Experiments for High-Throughput Automation in Human Metabolic Phenotyping
Source: Anal Chem. 2023 Jan 31;95(6):3147–52. doi: 10.1021/acs.analchem.2c04066 (PMC9933041; doi:10.1021/acs.analchem.2c04066)

Supporting Information

Optimization of Diffusion-Ordered NMR Spectroscopy Experiments for High-Throughput Automation in Human Metabolic Phenotyping

Nikita Harvey [1,2], Panteleimon G Takis [1,2], John C Lindon [3], Jia V Li [4] and Beatriz Jiménez [1,2]

[1] Section of Bioanalytical Chemistry, Division of Systems Medicine, Department of Metabolism, Digestion and Reproduction, Imperial College London, Burlington Danes Building, Hammersmith Hospital Campus, London, W12 0NN, UK

[2] National Phenome Centre, Department of Metabolism, Digestion and Reproduction, Imperial College London, IRDB Building, Hammersmith Campus, London, W12 0NN, UK; phenomecentre@imperial.ac.uk

[3] Section of Biomolecular Medicine, Division of Systems Medicine, Department of Metabolism, Digestion and Reproduction, Imperial College London, Burlington Danes Building, Hammersmith Hospital Campus, London W12 0NN, UK

[4] Section of Nutrition, Division of Digestive Diseases, Department of Metabolism, Digestion and Reproduction, Imperial College London, Commonwealth Building, Hammersmith Hospital Campus, London W12 0NN, UK

Index of Supporting Information

1	Pulse Sequences used for the 1D Analysis of Blood Samples	Page S2
2	Modified PROJECTED pulse sequence file for Bruker TopSpin	Page S4
3	Optimization of Experimental Parameters	Page S7
4	Automated acquisition and processing files for Bruker TopSpin 3	Page S12
5	Measured Metabolite Diffusion Coefficient Values	Page S13
6	Convection in the DOSY experiments	Page S16

1: Pulse Sequences used for the 1D Analysis of Blood Samples

In the analysis of complex biofluids including a mixture of small metabolites and macromolecules such as blood plasma, further techniques are used to produce edited spectra showing a subset of components (Beckonert et al., 2007; Dona et al., 2014). Relaxation editing in the form of the 1D Carr-Purcell-Meiboom-Gill (CPMG) experiment is routinely used to provide a small metabolite profile devoid of broad macromolecule peaks. Diffusion editing, conversely, is applied to produce a macromolecule profile which does not contain small metabolites signals. The separation of these overlapping components into independent spectra is key for the metabolic profiling protocol.

a) 1D-^{1}H general profiling spectrum on the blood plasma sample used in Figure 2 was acquired using the *noesygppr1d* sequence as described previously by Dona et al., 2014. Sharp signals correspond to small molecules or metabolites present in human blood plasma while broad signals belong to the macromolecule e.g., lipids, proteins, lipoproteins.

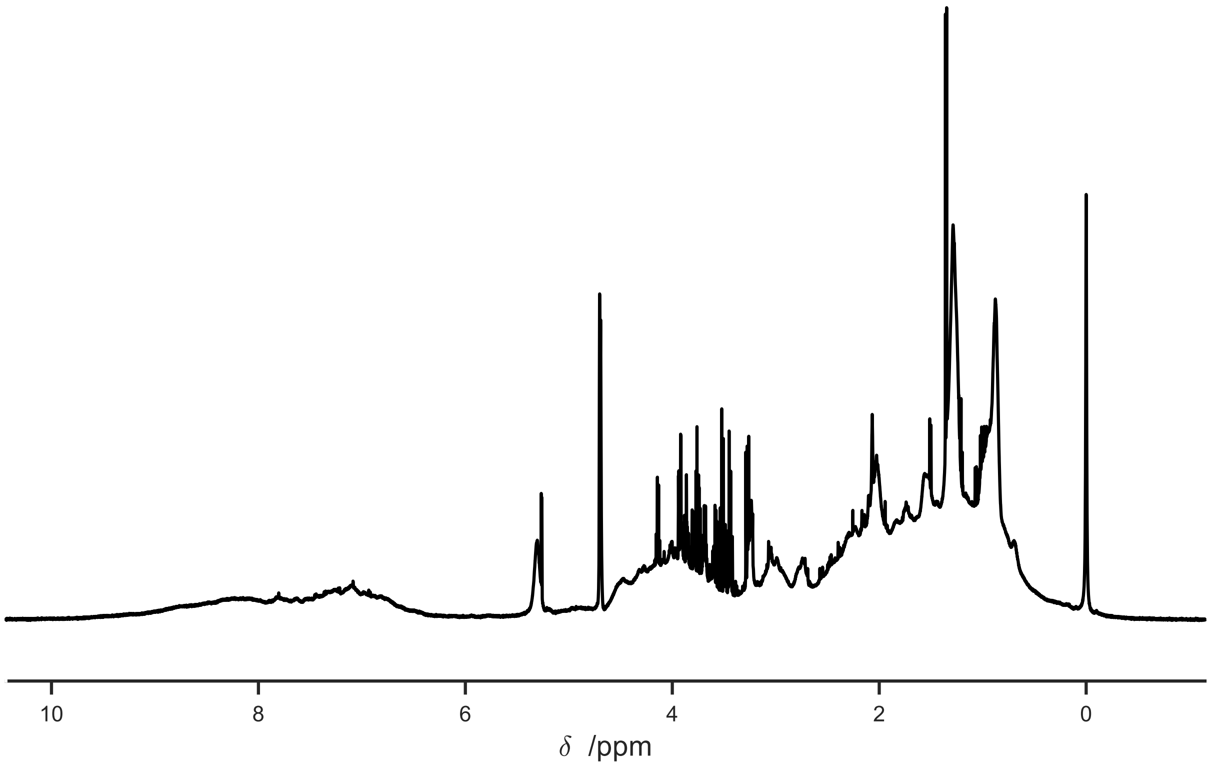

b) 1D CPMG spectrum was acquired on the same sample using the *cpmgpr1d* sequence as described by Dona et al., 2014. Due to relaxation editing, only small molecules are visible in this spectrum.

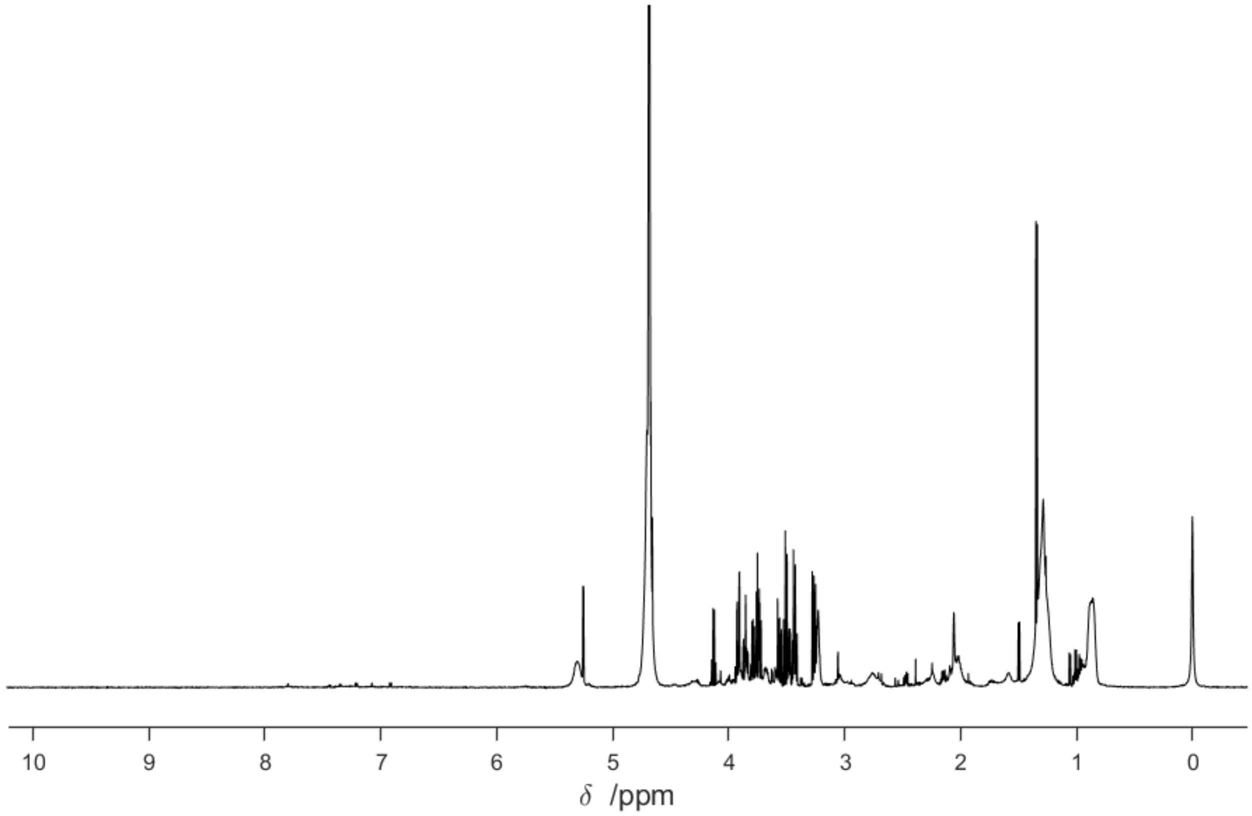

c) 1D diffusion-edited spectrum on the same sample was acquired using the ledbpgppr2s1d pulse sequence as described by Dona et al., 2014. Macromolecule signals only are visible in this spectrum.

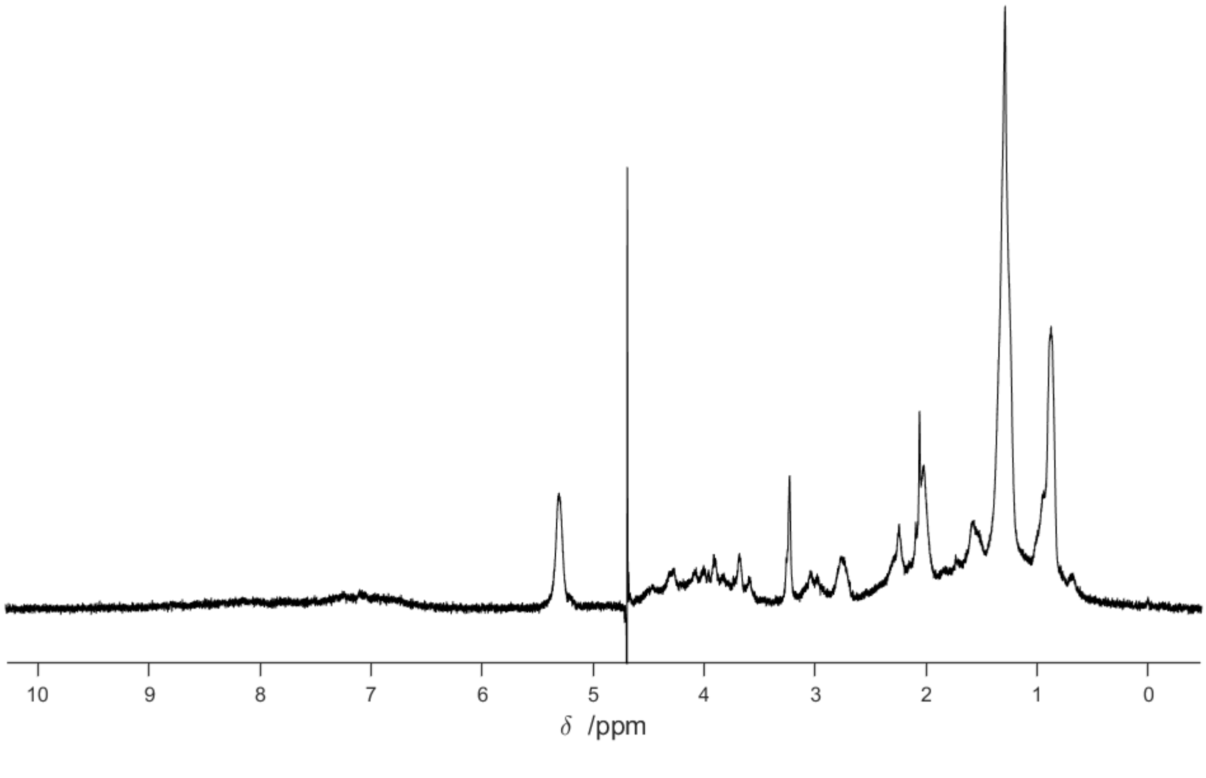

2: Modified PROJECTED pulse sequence

Schematic of modified PROJECTED pulse sequence adapted using Fig. 2 by Aguilar et al., 2014. $\pi/2$ = 90° hard pulse, π = 180° hard pulse, pr = presaturation pulse, Δ = unit diffusion time, τ indicates a constant defined primarily by Δ, RD = relaxation delay, n = number of loops, and ACQ = acquisition time. Modification consisted of addition of presaturation pulse and associated phase cycling. The PROJECTED pulse sequence was developed to suppress the effects of chemical exchange and *J*-modulation on measured diffusion coefficient values (Aguilar et al., 2014). The presaturation pulse and associated phase cycling was added by the authors.

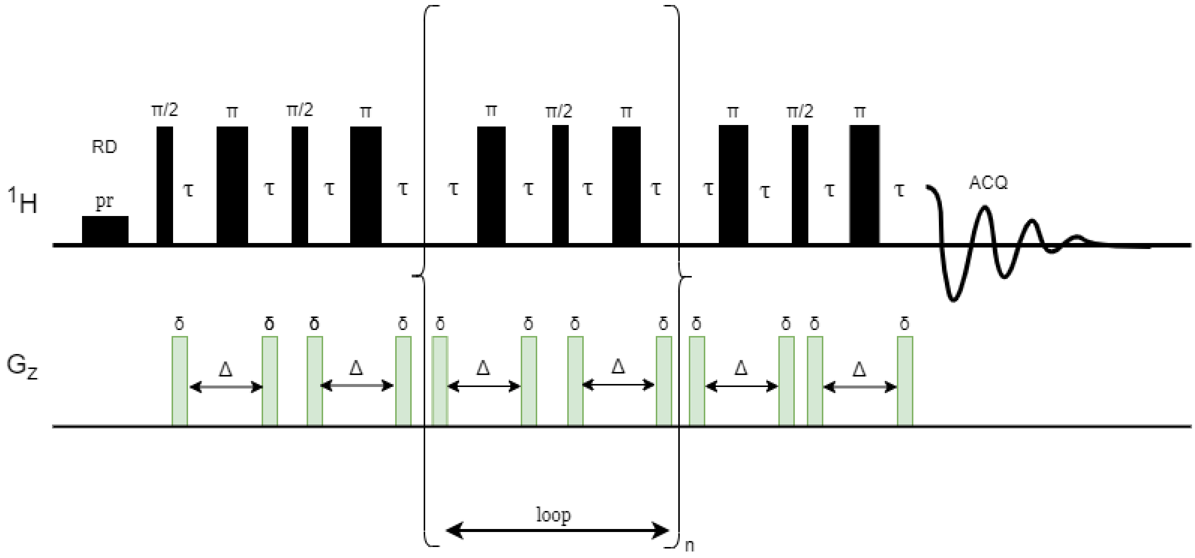


```
;/* The pulse sequence starts here */
;/*
;Juan A. Aguilar, Ralph W. Adams, Mathias Nilsson. Gareth A. Morris. Manchester.
;Development version, minimum phase cycle, subject to change.
;http://nmr.chemistry.manchester.ac.uk
;
;NOT convection compensated version.
;modified by BJ and NH to include presaturation */
;CLASS=HighRes
;$DIM=2D
;$TYPE=
;$SUBTYPE=
;$COMMENT=

#include <Avance.incl>
#include <Grad.incl>
#include <Delay.incl>

define list<gradient> diff=<Difframp>

"l5=l4-2"
"p2=p1*2"
"p3=p1"
"DELTA=d2*0.25-p30-d16"
"DELTA1=d2*0.25-p30-d16-50u"
```

```
"acqt0=-p1*2/3.1416"

1 ze
2 30m
d12 pl9:f1
d1 cw:f1 ph29
50u do:f1 UNBLKGRAD
d12 pl1:f1
p1 ph1 ; 1st 90
;;;;;;;;;;;;;;;;;;;;;;;;;;;;;;;; 1st cycle ;;;;;;;;;;;;;;;;;;;;;;;;;;;;;
50u UNBLKGRAD
p30:gp1*diff
d16
DELTA1
p2 ph2
DELTA
p30:gp1*diff
d16
p3 ph3
p30:gp1*diff
d16
DELTA
p2 ph2
DELTA
p30:gp1*diff
d16
;;;;;;;;;;;;;;;;;;;;;;;;;;;;;;;; 1st cycle ;;;;;;;;;;;;;;;;;;;;;;;;;;;;
;;;;;;;;;;;;;;;;;;;;;;;;;;;;;;;; LOOP ;;;;;;;;;;;;;;;;;;;;;;;;;;;;;;;;
3 p30:gp1*diff
d16
DELTA
p2 ph2
DELTA
p30:gp1*diff
d16
p3 ph3
p30:gp1*diff
d16
DELTA
p2 ph2
DELTA
p30:gp1*diff
d16
lo to 3 times l5
;;;;;;;;;;;;;;;;;;;;;;;;;;;;;;;; LOOP ;;;;;;;;;;;;;;;;;;;;;;;;;;;;;;;;
;;;;;;;;;;;;;;;;;;;;;;;;;;;;;;;; LAST cycle ;;;;;;;;;;;;;;;;;;;;;;;;;;;;
p30:gp1*diff
d16
DELTA
p2 ph2
DELTA
```

```
p30:gp1*diff
d16
p3 ph3
p30:gp1*diff
d16
DELTA
p2 ph2
DELTA1
p30:gp1*diff
d16
50u BLKGRAD
;;;;;;;;;;;;;;;;;;;;;;;;;;;;;;;;;;; LAST cycle ;;;;;;;;;;;;;;;;;;;;;;;;;;;;;;
go=2 ph31
30m mc #0 to 2 F1QF(igrad diff)
exit

ph1=0 2 ;90
ph2=1 ;180
ph3=1 1 3 3 ; 90 Refocusing
ph29=0
ph31=0 2 0 2 ; Receiver

;pl1 : f1 channel - power level for pulse (default)
;p1 : f1 channel - 90 degree high power pulse
;p30: gradient pulse (little DELTA*0.5)
;d1 : relaxation delay; 1-5 * T1
;d16: delay for gradient recovery
;d2 *0.5: unit diffusion time. 20-40 ms
;l4: number of cycles: 1 cycle= 2 diffusion time units
;NS: 1 * n
;DS: 1 * m
;td1: number of experiments
;FnMODE: QF
;use AU-program dosy to calculate gradient ramp-file Difframp
;//* The pulse sequence ends here */
```

3: Optimization of Experimental Parameters

a) Experimental Time

The traditional DOSY experiment was run with 256, 128, 64, 32, 16 and 8 increments to determine the number of increments required for the signal decay. It was found that increments of 16 or higher produced artefacts in the fitting of the signal decay to the Stejskal-Tanner equation. The experiment was also run with 64, 32, 16 and 8 scans to determine the sensitivity of the experiment. Automatic peak picking was performed on the initial increment of the 8 scan and 32 scan experiments and compared to the list of automatically picked peaks from the 1D-^{1}H general profile. A noise threshold of 5000 arbitrary units (au) was set with the smallest peak picked having an intensity of 102283 au. The 8 scan experiment contained 90% of the peaks from the general profile while the 32 scan experiment contained >99% of the peaks in the general profile. Dummy scans of 16, 8, 4 and 2 were tested and a minimum number of 8 dummy scans was determined to be required to avoid artefacts. The relaxation delay was tested at 1, 2, 3, 4 and 5 seconds and no noticeable increase in signal intensity was observed with relaxation delays above 2 seconds. Total experimental time was thereby reduced from 7 minutes 12 seconds (when using the standard 4 s delay) to 3 minutes 36 seconds.

The relaxation-edited experiment was run with 8, 16, 32 and 64 scans. It was found that the 8 scan experiment contained 75% of the signals peak picked from the 32 scan experiment. An increase in number of scans higher than 32 did not significantly increase the number of peaks picked.

The diffusion-edited experiment was also run with 8, 16, 32 and 64 scans. Whilst the signal in the aliphatic region had high intensity in the 8 scan experiment, the aromatic region of the spectrum was largely below the noise threshold in experiments with fewer than 64 scans. Signals from aromatic groups belonging to small molecules in proteinic samples such as blood plasma are broad and difficult to observe on the NMR spectrum due to the interaction with the macromolecules. Hence, for the routine analysis of small molecules, 8 scans will suffice as all signals from the aliphatic region are above the noise threshold. However, where the aromatic region of the macromolecule spectrum is of interest, 64 scans or higher are recommended for this experiment. Number of increments were also varied during optimisation (8, 16, 32, 64 increments). As the macromolecule diffusion coefficients are spread over a smaller range than the small molecule diffusion coefficients (0-1.5 x10^{-10} m^2/s as opposed to 2 – 15 x 10^{-10} m^2/s), 32 increments were necessary to obtain sufficient diffusion coefficient resolution to distinguish between different classes as in Table S1c.

b) Signal decay

The signal decay curve is determined by the diffusion delay and the gradient pulse length and affects the diffusion coefficient resolution. For the traditional DOSY experiment, diffusion delays of 50, 75,

100 and 150 ms were tested. Decrease of the diffusion delay from 75 ms (blue) down to 50 ms (red) resulted in a 4-fold increase in diffusion coefficient resolution as shown below. This 4-fold increase in resolution was calculated by measuring the signal linewidth in the diffusion dimension.

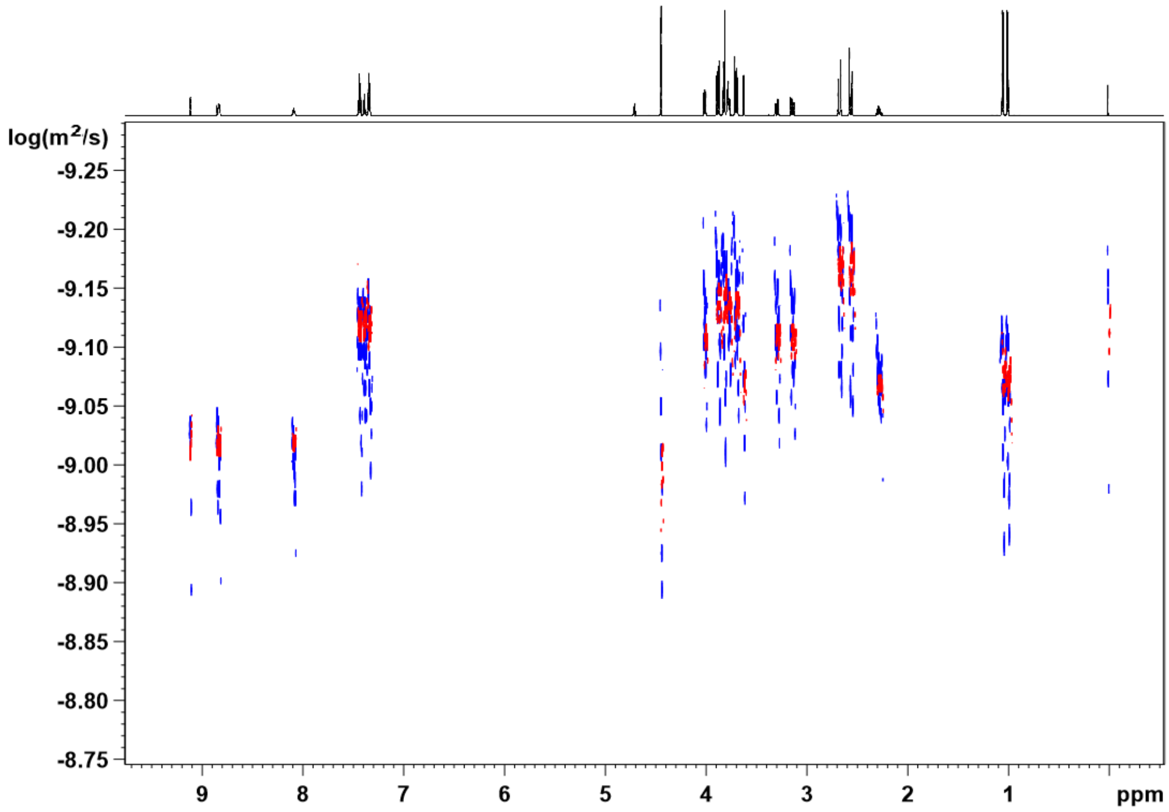

Gradient pulse lengths of 2.2, 1.85, 1.5 and 1 ms were tested and peak intensities picked from three metabolites to track signal decay. It was found that a gradient pulse length of 1.5 ms provided the optimal signal decay curve, reaching an almost complete signal decay of 97.2% at 95% gradient strength.

1D PROJECTED presat experiments were run on a pooled human serum sample with different unit diffusion times of 20 ms (blue), 25 ms (red), 30 ms (green), 35 ms (purple) and 40 ms (cyan) as shown below. Longer diffusion delays increase both noise and editing of macromolecules. A unit diffusion time of 30 ms was chosen to compromise between noise and macromolecule signal editing.

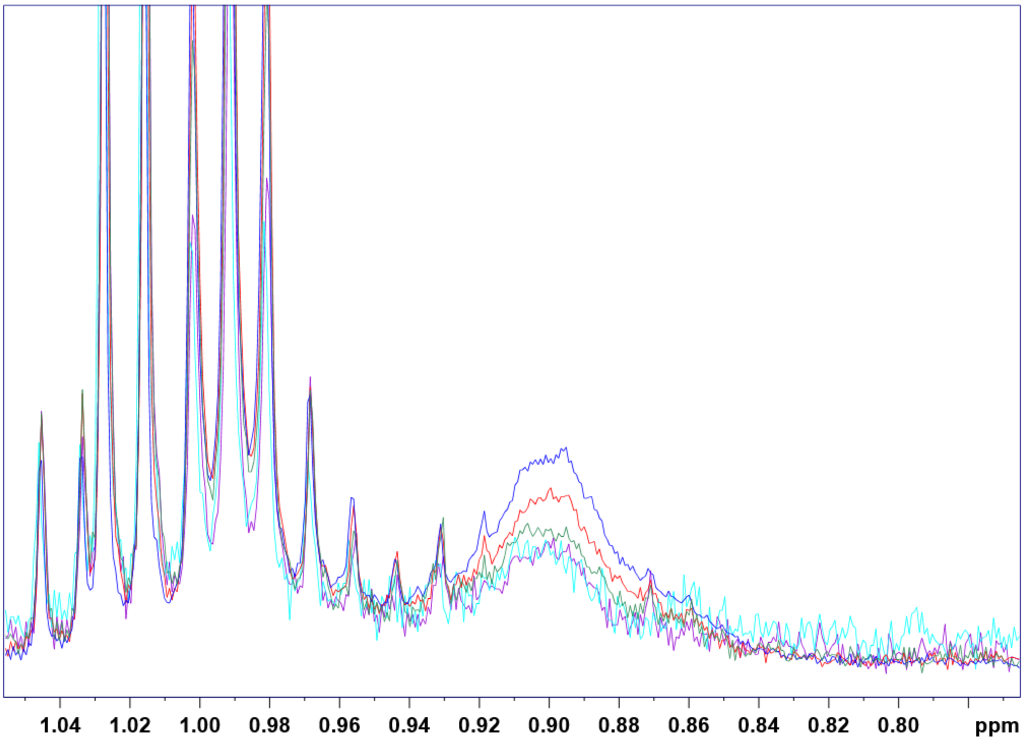

Gradient pulse lengths of 1, 0.8 and 0.6 ms were tested using the 1D PROJECTED presat experiment and peaks were picked from three metabolites to track the signal decay curve. A gradient pulse length of 0.8 ms was found to provide the optimal signal decay curve with a signal decay of 91.5% at the 95% gradient strength increment.

The diffusion-edited experiment required a longer diffusion delay to allow signal decay of the macromolecules. Diffusion delays of 150, 200, 300, 350, 400, 500, 600, 700, 800 and 900 ms were tested using the *ledgppr2s1d* pulse sequence at 95% gradient strength. The lipid and protein signals reached a signal decay of 84.2% with a diffusion delay of 350 ms. The gradient pulse length was kept consistent with the traditional DOSY experiment. However, the limits of the linear ramp used for the gradient strength were altered to provide diffusion editing. 1D DOSY experiments were run with a diffusion delay of 350 ms and gradient strengths of 5%, 20%, 25% and 95%. The spectrum run with 25% gradient retained only 5.5% small molecule signal intensity whilst retaining 82.2% of the macromolecule signal intensity (compared to the 5% gradient strength spectrum). The gradient ramp was therefore defined as 25-95% of the gradient strength.

Final optimised parameters as used with Bruker TopSpin 3 are laid out in the tables below.

Traditional DOSY experiment:

Parameter	TopSpin Parameter	Value
**Pulse Program**	PULPROG	ledbpgppr2s
**Points**	TD F2	16384
**Increments**	TD F1	8
**Scans**	NS	8
**Dummy Scans**	DS	8
**Sweep Width**	SW	15
**Receiver Gain**	RG	90.5
**Relaxation Delay**	D1	2 s
**Diffusion Delay**	D20	50 ms
**Spoil Gradient Length**	P19	600 µs
**Gradient Pulse Length**	P30	1.5 ms
**Gradient Power**	GPZ6	100%
**FID Baseline Mode**	BC_mod	qpol

PROJECTED presat:

Parameter	TopSpin Parameter	Value
**Pulse Program**	PULPROG	PROJECTED presat
**Points**	TD F2	16384
**Increments**	TD F1	8
**Scans**	NS	8
**Dummy Scans**	DS	8
**Sweep Width**	SW	15
**Receiver Gain**	RG	90.5
**Relaxation Delay**	D1	2 s
**Unit Diffusion Time**	D2	30 ms

**No. of Loops**	L4	14
**Spoil Gradient Length**	P19	600 μs
**Gradient Pulse Length**	P30	0.8 ms
**Gradient Power**	GPZ6	100%
**FID Baseline Mode**	BC_mod	qpol

Diffusion-edited DOSY:

**Parameter**	**TopSpin Parameter**	**Value**
**Pulse Program**	PULPROG	ledbpgppr2s
**Points**	TD F2	16384
**Increments**	TD F1	32
**Scans**	NS	8
**Dummy Scans**	DS	8
**Sweep Width**	SW	15
**Receiver Gain**	RG	90.5
**Relaxation Delay**	D1	2 s
**Diffusion Delay**	D20	350 ms
**Spoil Gradient Length**	P19	600 μs
**Gradient Pulse Length**	P30	1.5 ms
**Gradient Power**	GPZ6	100%
**FID Baseline Mode**	BC_mod	qpol

4: Automated acquisition and processing files for Bruker TopSpin 3

a) Automated acquisition

//Note that the text “dosy 5 95 %d l y n y” should be altered to “dosy 25 95 %d l y n y” in the case of the diffusion edited experiment.

```
int     td1;

XCMD("getprofpars");
FETCHPAR1("TD", &td1)
sprintf(text, "dosy 5 95 %d l y n y", td1);
XCMD(text)
QUIT
```

b) Automated processing

```
XCMD("setdiffparm")
XF2
STOREPAR("ABSG", 1);
STOREPAR("ABSF1", 100.0);
STOREPAR("ABSF2", -100.0);
ABS2
STOREDOSYPAR("DISPmin", -12.0)
STOREDOSYPAR("DISPmax", -8.0)
XCMD("dosy2d")
QUIT
```

5: Measured Metabolite Diffusion Coefficient Values

a) Diffusion coefficients obtained from mixture of metabolites in H_2O/D_2O (commercially available sample: Bruker, H144519_01). All the NMR peaks belonging to chemical groups from the same metabolite have the same diffusion coefficient and hence appear at the same position on the *y*-axis in the DOSY spectrum. The diffusion coefficient values of each compound are marked with a separate dotted line.

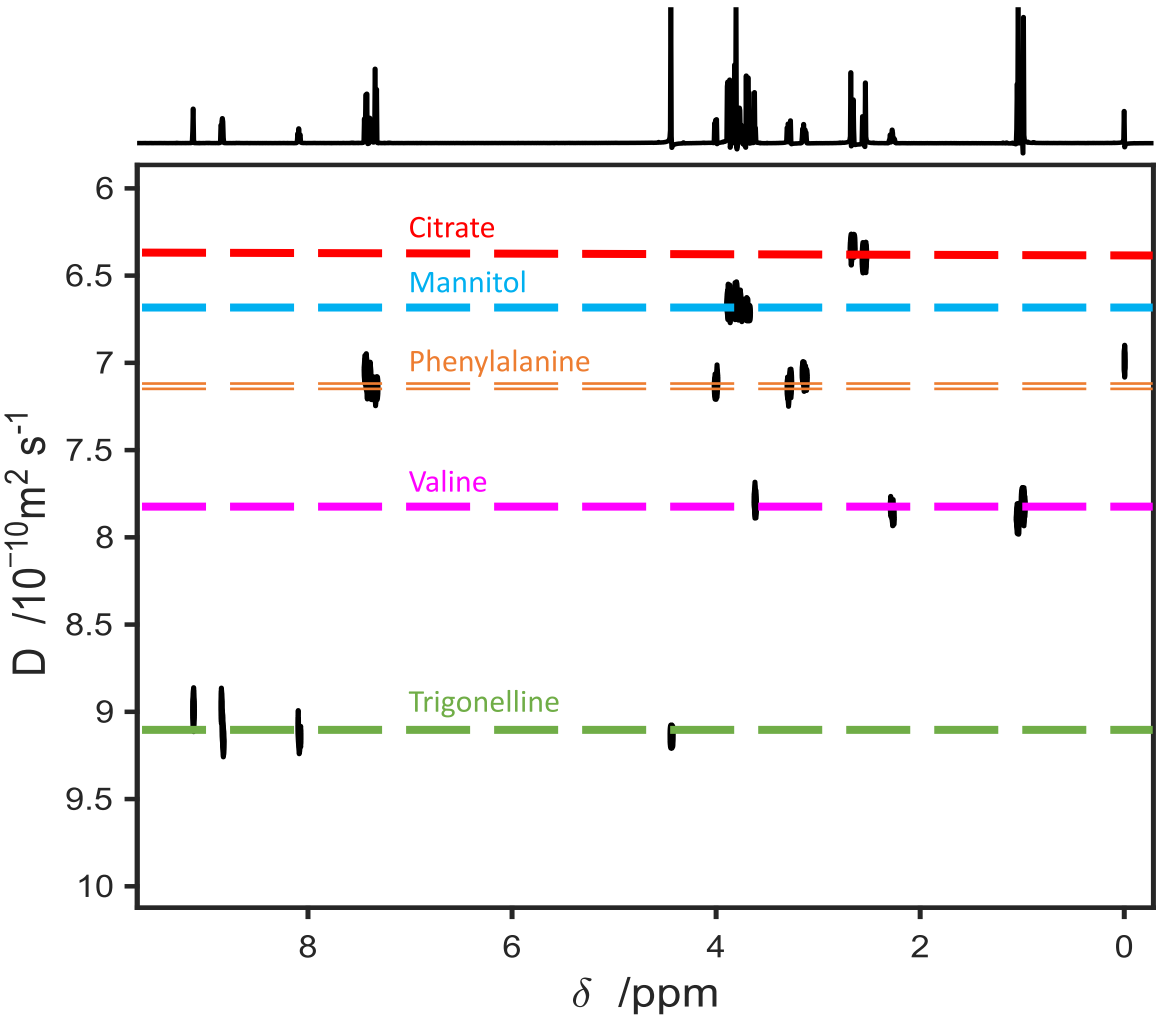

Metabolite	Average Diffusion Coefficient (x 10^{-10} m^2/s)
Valine	7.84 ± 1.30
Trigonelline	9.10 ± 0.10
Mannitol	6.68 ± 0.04
Citrate	6.37 ± 0.03
Phenylalanine	7.12 ± 0.09

b) Diffusion coefficients obtained from Human Urine sample:

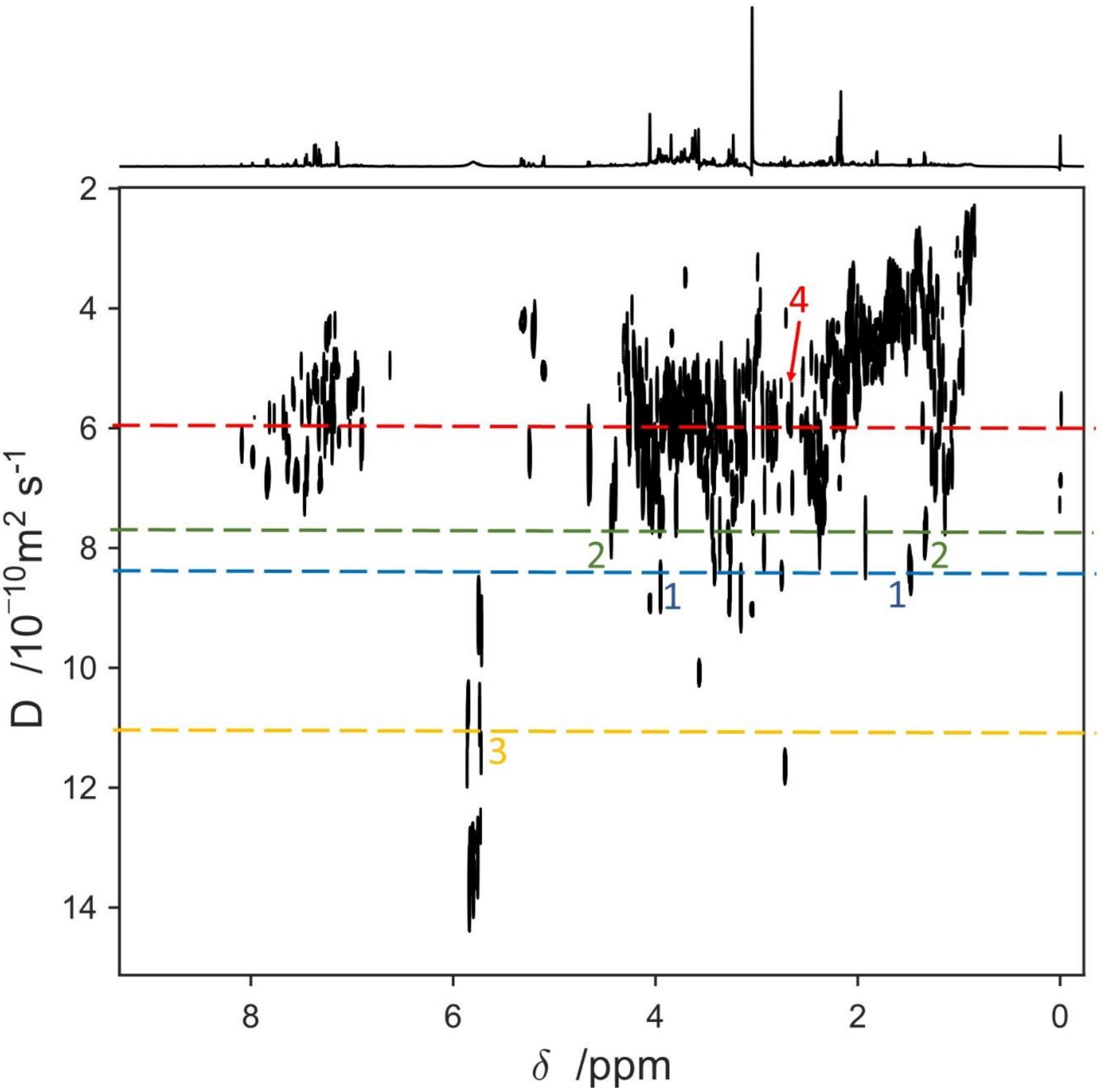

Fig. 1b label	Metabolite	Average Diffusion Coefficient (x 10^{-10} m^2/s)
1	Alanine	8.36 ± 0.09
2	Lactate	7.74 ± 0.11
3	Urea	11.14 ± 3.43
4	Citrate	5.94 ± 0.10

c) Diffusion coefficients obtained from Figure 2: Human Blood Plasma sample

Fig. 2 label	Metabolite	Average Diffusion Coefficient: PROJECTED (x 10^{-10} m^2/s)	Average Diffusion Coefficient: diffusion edited DOSY (x 10^{-10} m^2/s)
1	Alanine	9.52 ± 0.002	
2	Lactate	9.47 ± 0.19	
3	Valine	7.78 ± 0.52	
4	Glucose	7.20 ± 0.20	
5	Lipoproteins	0.737 ± 0.769	0.275 ± 0.116
6	Lipids		0.181 ± 0.189
7	Proteins		0.787 ± 0.418

6: Convection in the DOSY experiments

a) Comparison of DOSY experiments acquired on a human urine sample at 302.5 K in a room-temperature probe with variable temperature gas flow on (400 lph, red) and off (50 lph, blue) using the optimised traditional DOSY experiment. Spectra were acquired with a diffusion delay of 50 ms gradient pulse length of 2.2 ms, 8 scans, receiver gain of 362 (automatically set), 16384 points in the F2 plane and 8 points in the F1 plane. Contour levels have been kept equal between the experiments in this overlay and the 1D-^{1}H spectrum is shown above the DOSY spectra. Diffusion coefficient measurements are not significantly different between the two spectra, confirming the lack of convection effect for this set-up.

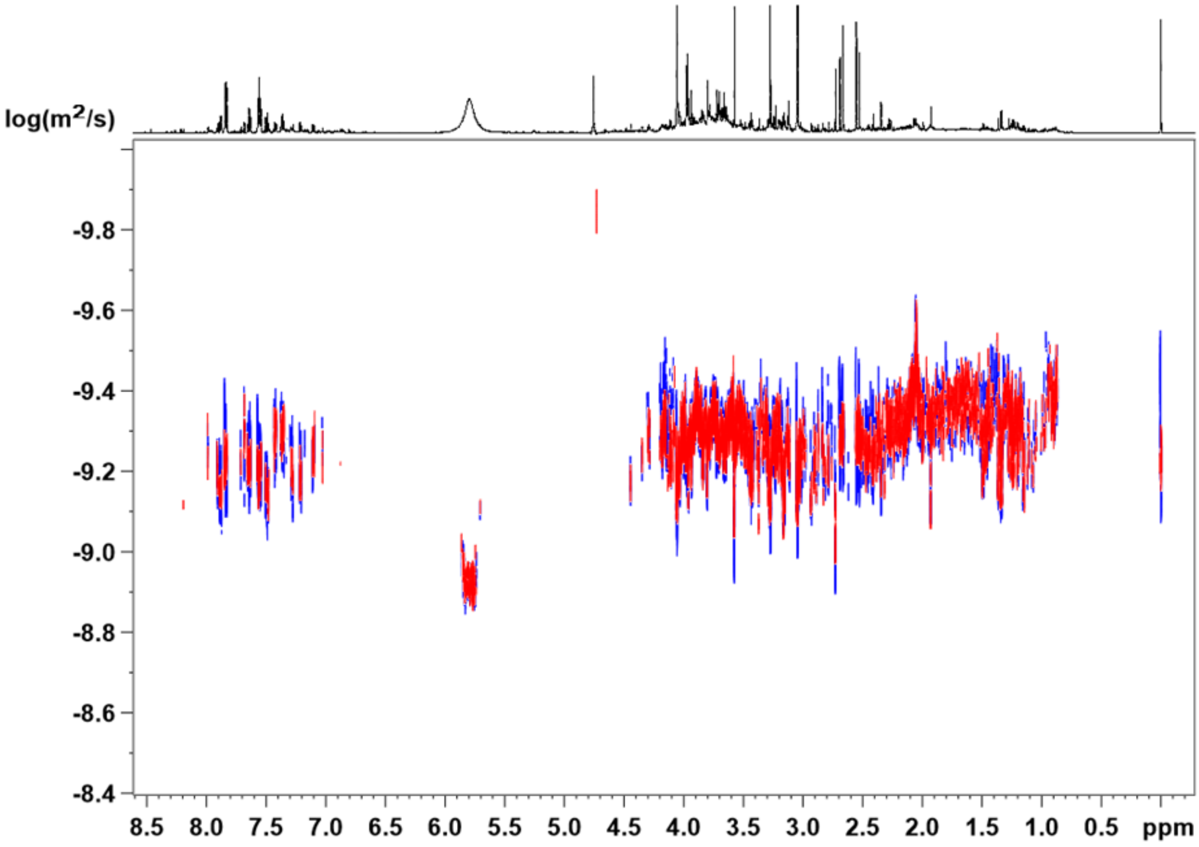

b) Comparison of PROJECTED presat (blue) and convection compensated PROJECTED presat (red) DOSY experiments on a spectrometer equipped with a room temperature probe. Spectra were acquired on the quantitation reference sample at 310 K with a unit diffusion time of 40 ms, gradient pulse length of 2.2 ms, 14 loop cycles, receiver gain of 90.5, 8 scans, 16384 points in the F2 plane and 8 points in the F1 plane. Contour levels have been kept equal between the experiments in this overlay. The 1D-^{1}H profile for this sample is shown above the DOSY spectra. Diffusion coefficient measurements are not significantly different between the two spectra despite the addition of the convection compensation elements to the pulse sequence as recommended (Aguilar et al, 2014).

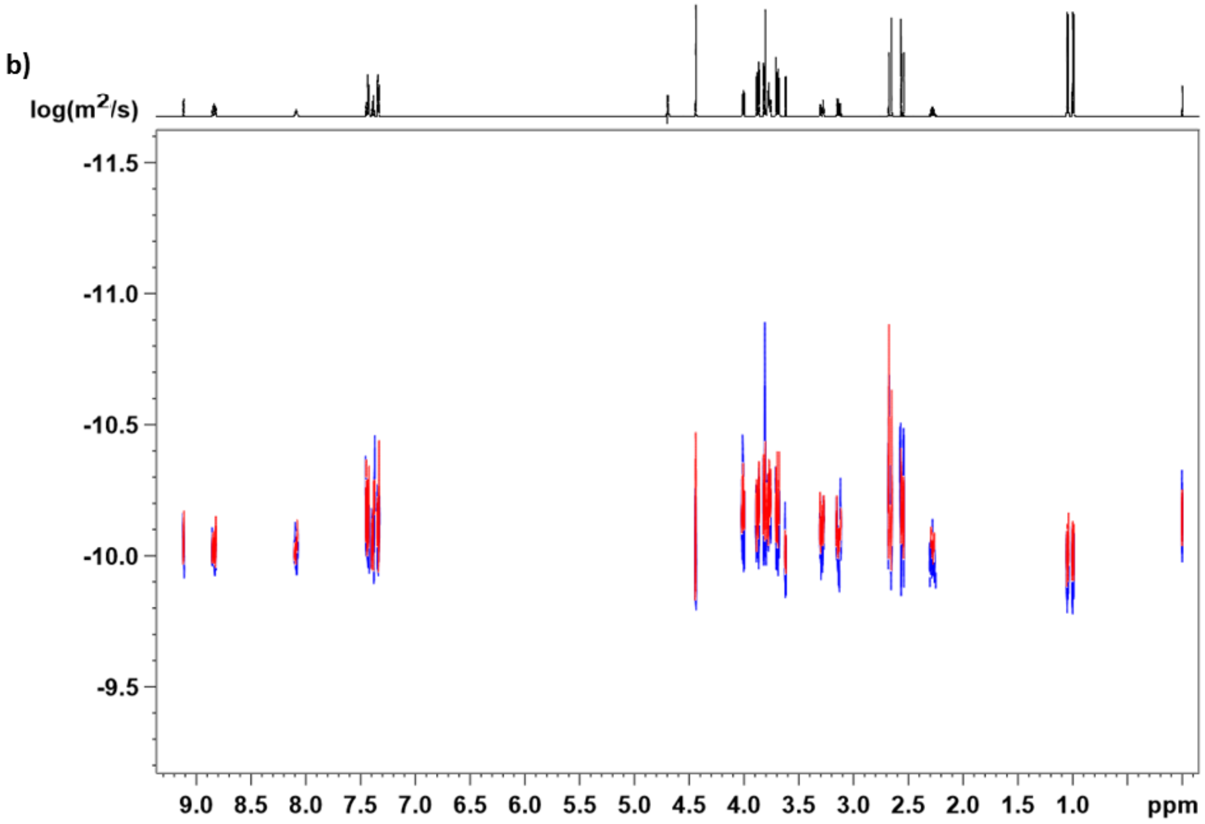

Supplement: Supplementary file 1 — ac2c04066_si_001.pdf [file ac2c04066_si_001.pdf]
